# Is it time to incorporate hands-on simulation into the cardiothoracic surgery curriculum?

**DOI:** 10.1093/icvts/ivab290

**Published:** 2021-10-26

**Authors:** Nabil Hussein, Alicja Zientara, Can Gollmann-Tepeköylü, Mahmoud Loubani

**Affiliations:** 1 Department of Congenital Heart Surgery, Yorkshire Heart Centre, Leeds General Infirmary, England, UK; 2 Royal Brompton and Harefield Hospital, England, UK; 3 Department of Cardiac Surgery, Medical University of Innsbruck, Austria; 4 Department of Cardiothoracic Surgery, Castle Hill Hospital, Cottingham, UK

**Keywords:** Simulation, Education, Cardiothoracic surgery, Pandemic, Curriculum

## Abstract

The COVID pandemic has had huge implications for training in cardiothoracic surgery. The reduction in training opportunities has led to concerns from trainees globally regarding the impact on their learning and their training progression. Surgical simulation is effective in the development of technical skills in cardiothoracic surgery with numerous examples of low and high-fidelity simulators. Despite this the incorporation of such methods into training curricula worldwide is seldom. Core fundamentals are required to successfully implement surgical simulation into training programmes, which includes; commitment from trainers, regular sessions and structured feedback. Few programmes have demonstrated the successful incorporation of surgical simulation and there is a growing acceptance of its place in the speciality. As we recover from this challenging period it may be the right opportunity to evolve how we train our current and future trainees by incorporating hands-on simulation as a fundamental part of the cardiothoracic curriculum.

After more than a year, we continue to see the effects of the coronavirus disease 2019 pandemic on society. The impact has been felt strongly within the CTS community and has had huge implications for training. The reasons for this are multifactorial, ranging from reductions in elective operative work and critical-care capacity, redeployment of trainees into intensive care and medical specialities, to social distancing protocols limiting operative and clinical exposure [1–3]. The reduction in training opportunities has led to concerns from trainees globally regarding both the impact on their learning and their ability to progress through their respective training pathways [1, 3].

Our speciality has adapted to these challenges and rapidly transitioned to online platforms to continue the delivery of clinical and academic excellence. This change has been well received worldwide by trainees who are keen to continue this platform of education; however, it is primarily didactic in nature [3]. Consequently, the ability to develop technical surgical skills, which is a fundamental part of CTS training, has been substantially hindered.

Surgical simulation has shown to be effective in the development of technical skills across multiple specialities including CTS [4–6]. This platform promotes the concept of deliberate practice whereby trainees can repeatedly rehearse a particular task in the absence of perioperative pressures [7]. An essential component is the provision of feedback, which allows trainees to identify their weaknesses and focus their training. This iterative cycle is likely to streamline the acquisition of technical skills in preparation for reality.

CTS simulation has been used across the speciality and can be broadly categorized based on its modality [8]. It includes cadaveric/animal and synthetic platforms (i.e. from simple physical three-dimensional printed/moulded models to virtual and augmented reality). These platforms can be further categorized on the basis of the fidelity of the simulators, which relates to how closely the simulation reflects reality (i.e. task simulators vs team-based scenarios). High-fidelity simulators closely resemble an actual task in a real environment and exist within various fields of CTS [8]. They include trainable basic skills (i.e. establishing cardiopulmonary bypass) to more advanced situational training such as emergency scenario management. In addition to improving the acquisition and retention of both technical and non-technical skills, it is hypothesized that these simulated skills in CTS will translate into the clinical setting with a reduction in costs and medical errors and will improve overall performance.

Although the incorporation of regular simulation modalities into CTS training programmes is sparse, there are examples where it has been fully integrated into the curricula [6, 9, 10]. For example, the UK national CTS programme has a portfolio of at least 10 curriculum-aligned training courses that utilizes both low- and high-fidelity simulators that cover a wide spectrum of technical and non-technical skills [9]. A similar programme exists in the United States, which combines didactic sessions with high-fidelity simulations designed to improve resident performance in a variety of speciality-specific skills [10]. A similar theme has also been demonstrated in congenital heart surgery with the incorporation of a monthly Hands-On Surgical Training programme in Toronto, Canada, whereby trainees rehearse complex congenital heart procedures on three-dimensional printed models and receive objective feedback on their performance [6]. These examples, among others, have shown an increased acceptance of simulations that has been driven by improved teaching behaviours, the utilization of technology and the growth of objective evidence demonstrating its value. We are now seeing this approach mature into standardized national training programmes, which include simulation as a fundamental component.

Core fundamentals are required to successfully implement surgical simulation into training programmes. This type of programme requires commitment from trainers and institutions to engage and develop curricula by sharing responsibility to manage the extra workload. Regular training sessions should be organized during protected teaching times to minimize disruptions, and structured feedback should occur regularly to focus training. As evidence grows demonstrating the objective improvements in technical performance, training programmes should actively seek to fund such initiatives, collaborate and share developments and strive to incorporate such methods into their respective curricula. However, further work is required to demonstrate the financial costs related to incorporating simulation methods into training curricula and whether simulation impacts the length of clinical training. Moreover, there may be a role for simulation in the re-training of skills in established surgeons, however studies are required to validate this as a potential use of simulation.

With the transition to online platforms to deliver didactic teaching, the same goals can be achieved with simulation [6]. Equipment can be delivered to trainees beforehand, and trainers can demonstrate the simulated task using video conferencing. Trainees can then stream their attempts via focused online classrooms under the supervision of trainers who can provide continuous feedback. This platform also allows one to record performances, which can then be retrospectively analyzed to give objective feedback to the trainee. During a time where social distancing protocols have limited large training sessions, this platform provides a safe alternative while maintaining the advantages of simulation.

It is well appreciated that simulation does not replace real-life learning at the operating table and that surgical performance includes a plethora of skills outside of technical efficiency. Although it has not yet been convincingly demonstrated within CTS that simulation translates to improvements in the intraoperative arena, randomized controlled trials in other specialities have shown that simulation leads to improvements in real-life performance [4, 5].

Throughout this challenging period, our speciality has adapted and thrived in delivering material to trainees so that they can continue their academic and clinical training. However, concern is growing regarding the impact the pandemic will have on the development of technical skills by the trainees. This concern is further compounded by the increasing subspecialization of CTS in the areas of minimally invasive/robotic surgery and the growth of interventional procedures, which will potentially limit surgical training. Simulation has been adopted within our speciality; however, its implementation globally is limited. As we recover from this relentlessly difficult period, it may be time to use this period as an opportunity to evolve how we train our current and future trainees by incorporating hands-on simulation as a fundamental part of the CTS curriculum.

**Conflict of Interest:** None.
