# Corrigendum To ‘Is it time to incorporate hands-on simulation into the cardiothoracic surgery curriculum?’ [Interact CardioVasc Thorac Surg 2021; https://doi.org/10.1093/icvts/ivab290]

**DOI:** 10.1093/icvts/ivab347

**Published:** 2021-12-13

**Authors:** Nabil Hussein, Alicja Zientara, Can Gollmann-Tepeköylü, Mahmoud Loubani

**Affiliations:** 1 Department of Congenital Heart Surgery, Yorkshire Heart Centre, Leeds General Infirmary, England, UK; 2 Royal Brompton and Harefield Hospital, England, UK; 3 Department of Cardiac Surgery, Medical University of Innsbruck, Austria; 4 Department of Cardiothoracic Surgery, Castle Hill Hospital, Cottingham, UK

In the original version of this article, the last name of author Can Gollmann-Tepeköylü had been misspelled. This has now been corrected above and in the full article online.

